# Succinobucol’s New Coat — Conjugation with Steroids to Alter Its Drug Effect and Bioavailability

**DOI:** 10.3390/molecules16119404

**Published:** 2011-11-10

**Authors:** Ondřej Jurček, Satu Ikonen, Lucie Buřičová, Martina Wimmerová, Zdeněk Wimmer, Pavel Drašar, Jan Horníček, Adéla Galandáková, Jitka Ulrichová, Erkki T. Kolehmainen

**Affiliations:** 1 Department of Chemistry, FI-40014, University of Jyväskylä, P.O. Box 35, Finland; Email: satu.m.ikonen@gmail.com (S.I.); erkki.t.kolehmainen@jyu.fi (E.T.K.); 2 Isotope Laboratory, Institute of Experimental Botany, Academy of Sciences of the Czech Republic, Vídeňská 1083, 14220, Prague 4, Czech Republic; Email: lucie.buricova@gmail.com (L.B.); martina.wimmerova@vscht.cz (M.W.); wimmer@biomed.cas.cz (Z.W.); 3 Institute of Chemical Technology, Prague, Technická 5, 16628, Prague 6, Czech Republic; Email: pavel.drasar@vscht.cz; 4 Department of Spectroscopy and Physical Organic Chemistry, Institute of Organic Chemistry and Biochemistry, Academy of Sciences of the Czech Republic, 16610, Prague 6, Czech Republic; Email: hornicek@uochb.cas.cz; 5 Palacký University in Olomouc, Institute of Translational and Molecular Medicine, Hněvotínská 3, 77515, Olomouc, Czech Republic; Email: Alfa.Baba@seznam.cz (A.G.); JitkaUlrichova@seznam.cz (J.U.)

**Keywords:** succinobucol, phytosterol, atherosclerosis, cholesterol, probucol

## Abstract

Synthesis, detailed structural characterization (X-ray, NMR, MS, IR, elemental analysis), and studies of toxicity, antioxidant activity and bioavailability of unique potent anti-atherosclerotic succinobucol-steroid conjugates are reported. The conjugates consist of, on one side, the therapeutically important drug succinobucol (4-{2,6-di-*tert*-butyl-4-[(1-{3-*tert*-butyl-4-hydroxy-5-(propan-2-yl)phenyl]sulfanyl}ethyl)sulfanyl]phenoxy}-4-oxo-butanoic acid]) possessing an antioxidant and anti-inflammatory activity, and on the other side, plant stanol/sterols (stigmastanol, β-sitosterol and stigmasterol) possessing an ability to lower the blood cholesterol level. A cholesterol-succinobucol prodrug was also prepared in order to enhance the absorption of succinobucol through the intestinal membrane into the organism and to target the drug into the place of lipid metabolism—The enterohepatic circulation system. Their low toxicity towards mice fibroblasts at maximal concentrations, their antioxidant activity, comparable or even higher than that of ascorbic acid as determined by direct quenching of the DPPH radical, and their potential for significantly altering total and LDL cholesterol levels, suggest that these conjugates merit further studies in the treatment of cardiovascular or other related diseases. A brief discussion of succinobucol’s ability to quench the radicals, supported with a computational model of the electrostatic potential mapped on the electron density surface of the drug, is also presented.

## 1. Introduction

Coronary artery diseases still remain one of the major causes of morbidity and mortality in the developed world. The pathogenesis of atherosclerosis lies in abnormalities in the lipoprotein metabolism leading to pathological interactions with blood vessel walls and a release of inflammatory components, which further aggravate the disease conditions. The accumulation of lipids within arteries remains the initial impulse for the pathogenesis of atherosclerosis; however, both oxidative stress and inflammation are considered to play crucial roles in this process [1,2,3,4].

Many large-scale clinical trials have documented a decrease in cardiovascular morbidity and mortality by lowering the LDL-cholesterol level by several therapeutic (hypolipidemics) and/or life style modification (diet, physical exercise) strategies in patients with multiple risk factors. Among the hypolipidemics our attention is directed to fatty acid esters of plant stanols/sterols and [4-{2,6-di-*tert*-butyl-4-[(1-{3-*tert*-butyl-4-hydroxy-5-(propan-2-yl)phenyl]sulfanyl}ethyl)sulfanyl]phenoxy}-4-oxo-butanoic acid] (succinobucol) a derivative of probucol (4,4'-[propane-2,2-diylbis(thio)]bis(2,6-di-*tert*-butylphenol)).

The addition of lipophilic fatty acid esters of plant stanol/sterol (cholesterol absorption blockers) to a diet significantly lowers serum total and LDL-cholesterol. This cholesterol malabsorption does not consistently affect HDL-cholesterol or triglyceride levels [5,6,7]. The esterified dietary plant stanols/sterols are known to be hydrolyzed in the upper part of the small intestine into free stanols/sterols and fatty acids in the same way as dietary lipids. The free plant stanols/sterols are then active in altering the level of absorbed and distributed cholesterol by various mechanisms. Saturated plant stanols appear to modify the cholesterol serum level more effectively than unsaturated plant sterols [5,6,7,8,9,10,11,12].

Hypolipidemics have been developed for targeting both the cholesterol metabolism and anti-inflammatory/antioxidant processes. Among them, probucol potentially impacts on atherosclerosis and related disorders via a range of biological activities, including its ability to affect lipid metabolism, exert an anti-inflammatory and antioxidant activity, and maintain the endothelial cell function. Unfortunately, during clinical trials, probucol was found to prolong the cardiac cellular repolarization, resulting in a prolongation of the QT interval and an attendant risk of potentially fatal cardiac arrhythmias. Probucol also lowers the HDL-cholesterol and only moderately decreases the LDL-cholesterol level [13,14]. In search for agents that share the anti-atherosclerotic activities but not the strong deleterious effects of probucol, succinobucol (AGI-1067), the monosuccinate ester of probucol, was discovered [13,15]. Succinobucol underwent phase III clinical trials to determine its effect on atherosclerotic endpoints; nevertheless, these results have not provided consistent data supporting strong cardioprotective effects (e.g., for an imbalance of cholesterol levels). Its studies still continue due to significant antihyperglycemic effects found. Its impact on type 2 diabetes is currently evaluated in additional phase III clinical studies [15,16].

Combined therapy approaches have been described, e.g., stanol fatty acid esters were combined with statins [17] or statins were combined with probucol [18]. However, no studies have been published describing combinations of probucol with plant stanols/sterols. The conjugation of both into a single entity offers a possibility of combining the biological effects described above. This could open the way to a positive impact on the metabolism of lipoproteins, added antioxidant and anti-inflammatory activity, and regulated drug distribution, ultimately leading to improved effects in the treatment and/or regression of atherosclerosis. Cholesterol has been used in conjugation with different drugs to increase their transport through the intestinal wall [19]. Therefore a model cholesterol-succinobucol conjugate was also prepared.

The protection from the atherosclerosis and the treatment of the disease are complex problems with many unknowns, and so are the modes of action of novel drugs and their metabolism. Many studies concerning probucol and succinobucol have been published [13,14,15,20,21,22,23]. Starting with these conjugates, a long pathway to discover their real potential is ahead. Studies of their toxicity, their bioavailability, their stability in the organism, their antioxidant activity, as well as solving of their conjugate (ligand) crystal structure for computer-aided investigations of their binding to receptor sides, are just a few of the initial steps to discover the full potential of the compounds reported in this work.

## 2. Results and Discussion

### 2.1. Synthesis

**Scheme 1:**
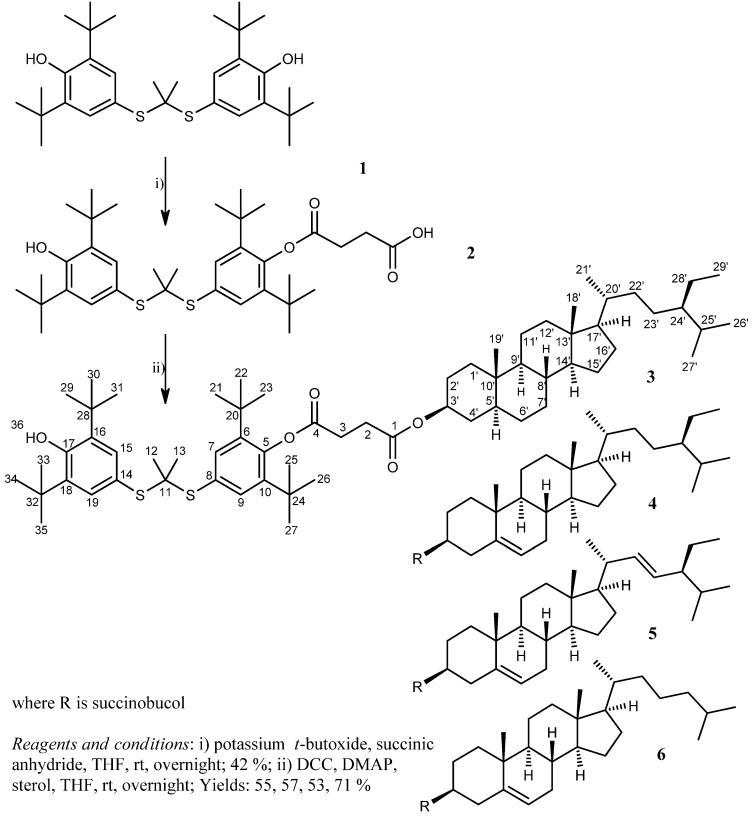
The preparation of succinobucol (**2**) and succinobucol-stanol/sterol conjugates **3**–**6**. The numbering of the carbon atoms of the compounds **2** and **4**–**6** can be derived from conjugate **3**.

In the initial work, one of the hydroxyl groups of probucol (**1**) was esterified by succinic acid anhydride in the presence of potassium *t*-butoxide as a deprotonating agent to give succinobucol (**2**). The carboxylic functionality of **2** was esterified under the conditions of Steglich esterification by different steroid alcohols. Succinobucol conjugates with stigmastanol (**3**), β-sitosterol (**4**), stigmasterol (**5**) and cholesterol (**6**) were obtained in good yields (Scheme 1).

### 2.2. Crystal Structure Determination

**Figure 1:**
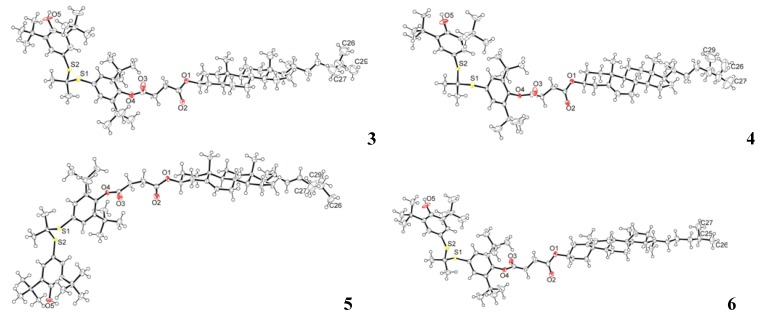
Molecular structures of **3**–**6** in the crystalline state. Only one molecule from each asymmetric unit is shown for clarity.

The compounds **3**–**6** were crystallized from a mixture of diethyl ether and acetonitrile at ambient temperature as colorless single crystals of X-ray quality. All compounds **3**–**6** crystallized in the triclinic spacegroup *P*1 (No.1) with either two (compounds **3**, **4**, **6**) or four (compound **5**) crystallographically independent molecules in the asymmetric unit. Molecular structures of one of the crystallographically independent molecules of each compound in the crystalline state are shown in Figure 1. More detailed results of the crystallographic study of these compounds have been reported elsewhere [24].

### 2.3. Toxicity Tests

**Figure 2:**
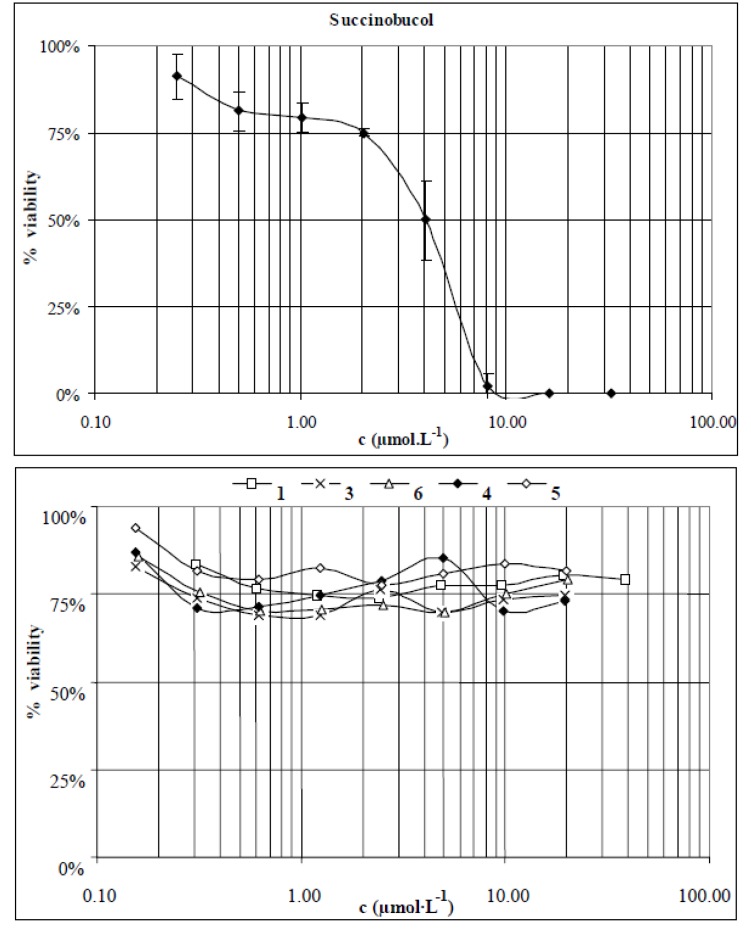
Toxicity profiles of succinobucol and compounds **1**, **3**, **4**, **5** and **6**.

Mouse fibroblasts the Balb/c 3T3 cell line were used as a standard model in the testing of toxicity of the compounds **1**–**6**. The cells were exposed to various concentrations of the studied compounds (0.16–40 µmol·L^−1^) and incubated for 24 h. After the treatment the cells were examined for signs of toxicity microscopically and by the MTT test. Of all the studied compounds only succinobucol (**2**) showed a toxic effect on the cells (IC_50_ 4 ± 1 µmol·L^−1^). Probucol (**1**) and conjugates **4** and **5** were not toxic at the highest soluble concentrations of 19 and 5 µmol·L^−1^, respectively. Conjugates **3** and **6** showed slight cytotoxicity, with cell viability at the highest soluble concentration of 5 µmol·L^−1^ being 70% for both compounds. The toxicity profiles obtained for the studied compounds are presented in Figure 2.

### 2.4. Stability Tests of Succinobucol and Conjugates ***3–6*** under Acidic Conditions

The tested compounds (2 mg) were dissolved in an aqueous solution of hydrochloric acid (pH = 1, 1 mL) and stirred at 37 °C to partially mimic the gastric environment. The samples were monitored by TLC for possible changes of a sample location or splitting on the plate signaling decomposition. The samples were deposited on plates in the intervals of 3, 10, 20, 30, 70, 180 min. No observable decomposition of the compounds was detected within this period.

### 2.5. 1,1-Diphenyl-2-picrylhydrazyl Radical (DPPH) Scavenging Activity and its Mechanism of Action

The EC_50_ value (Efficient Concentration = [(mol/L)AO/(mol/L)DPPH] represents the amount of antioxidant necessary to decrease the initial DPPH concentration by 50%. For reasons of clarity we speak in terms of 1/EC_50_ or the antiradical power (ARP): the larger the ARP, the more efficient the antioxidant is [25]. It can be concluded from Table 2 that the conjugates **5** and **6** have an ARP similar to that of ascorbic acid, while the remaining studied compounds, the parental compounds **1** and **2** and the conjugates **3** and **4**, have a higher ability to scavenge the DPPH radical and thus a higher ARP than ascorbic acid.

**Table 2:** Radical scavenging activity of test compounds **1**–**6**.

Compound	Ascorbic acid	1	2	3	4	5	6
EC_50_	0.27	0.20	0.17	0.20	0.14	0.27	0.26
ARP	3.7	5.0	5.9	4.9	7.1	3.7	3.8

**Figure 3:**
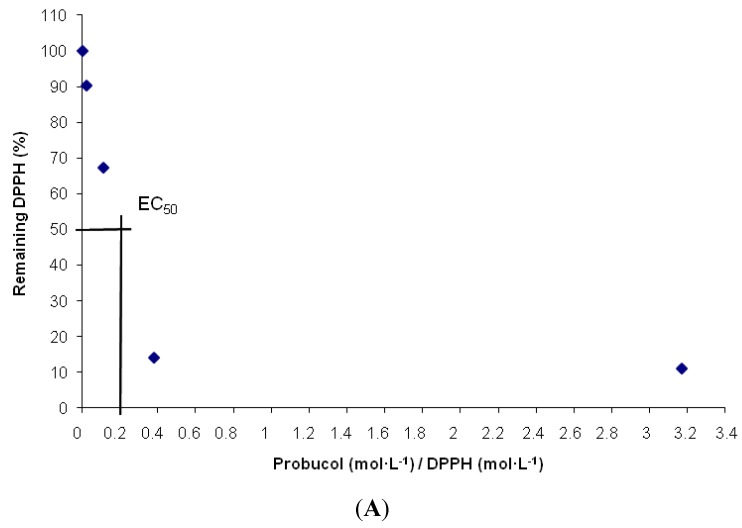

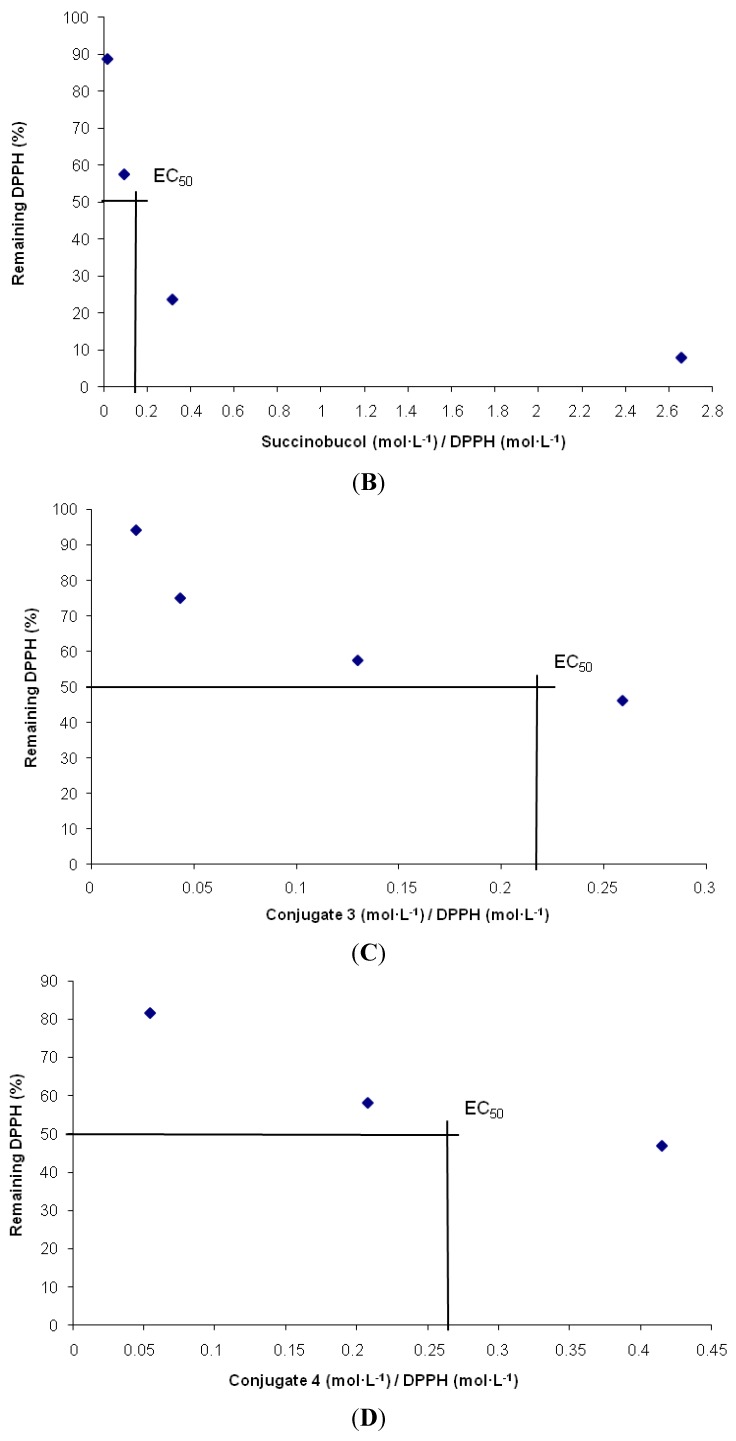

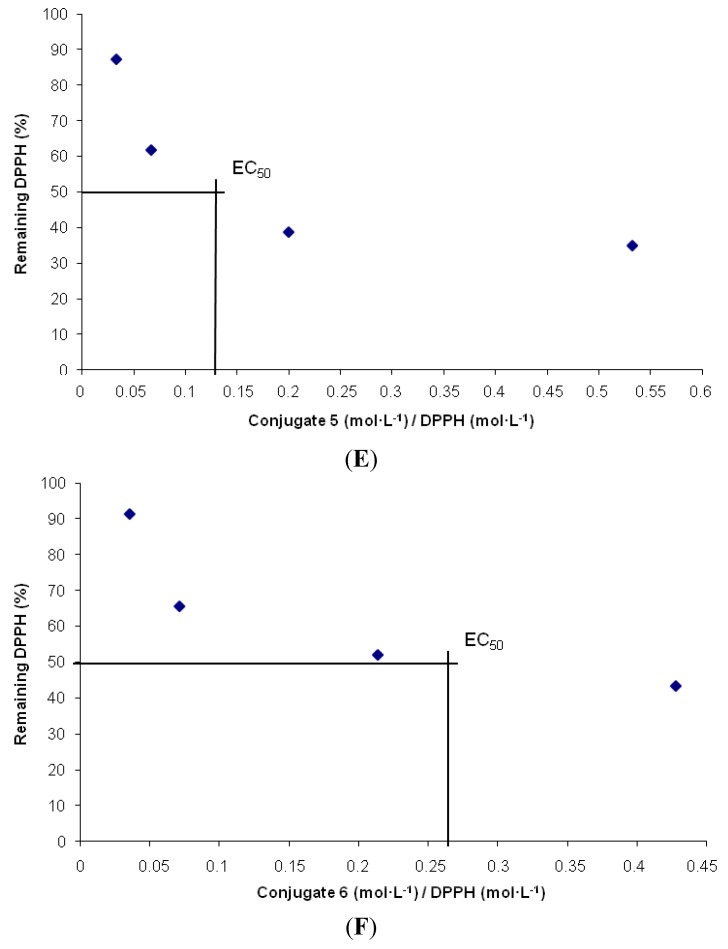
Antioxidant activity profiles. (**A**) probucol; (**B**) succinobucol; (**C**) Conjugate 3; (**D**) Conjugate 4; (**E**) Conjugate 5; (**F**) Conjugate 6.

The antioxidant activity profiles obtained for the studied compounds are presented in Figure 3.

**Figure 4:**
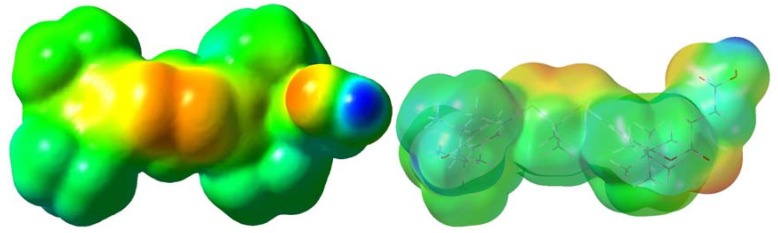
Electrostatic potential on the molecule surface of a succinobucol molecule in methanol (red indicates negative, blue indicates positive and green indicates neutral electrostatic potential).

From the comparison of results of probucol measurements with other compounds from the studied group, one can conclude that their activity is not dependent on the number of available phenolic hydroxyl groups, but other features should be considered. Sulfur atoms in organic compounds are known to be active in quenching of radicals [26,27]. It was also observed by the group of Stocker that the sulfur atoms, rather than the phenolic moieties of probucol (or succinobucol), may play the key role in antioxidant activity, and thus may be responsible for the antiatherogenic and antirestenotic protection [23]. We assume that in our case also the sulfur atoms are responsible for the antioxidant activity. This was also suggested by the computed electrostatic potential mapped on the electron density surface of the succinobucol molecule in methanol (all computational operations were performed with the Gaussian 09 Rev A.02 software package. The geometry optimizations were performed using the B3LYP functional with the 6-311G** basis set. The Gaussian CPCM model was used to provide solvent effects). The negative atomic polar tensor (APT) charge for oxygen in the free phenolic hydroxyl group is −0.64 (reduced by the value of a hydrogen charge), compared to sulfur atom’s, −0.03 and −0.09, respectively. Even though the oxygen carries a higher negative charge, it may be less susceptible to electron/radical donation, because of the shielding by bulky *t*-butyl groups, contrary to the relatively unshielded sulfur atoms (Figure 4). 

## 3. Experimental

### 3.1. Chemistry

NMR: ^1^H and proton decoupled (waltz-16) ^13^C-NMR spectra in dilute CDCl_3_–solutions at 303 K were run on a Bruker Avance DRX 500 NMR spectrometer equipped with a 5 mm diameter broad band inverse probehead working at 500.13 MHz for ^1^H and at 125.76 MHz for ^13^C, respectively. ^1^H chemical shifts were referenced to the trace signal of CHCl_3_ (7.26 ppm from int. TMS) and ^13^C chemical shifts to the center peak of the solvent signal (77.00 ppm from int. TMS). The assignments of the individual ^1^H and ^13^C signals were carried out by comparing them with NMR data of parental compounds published in the literature [28,29,30], by heteronuclear 2D experiments PFG ^1^H, ^13^C HMQC and HMBC, and supported by software ACD/ChemSketch C+H NMR Predictors and DB (Product version 10.04). MS: In order to ascertain the molecular weights of compounds, ESI-TOF mass spectra were recorded on a Bruker instrument model Micromass LCT operated by software Masslynx version 3.5. The compounds were dissolved in methanol (HPLC grade) and diluted to appropriate concentrations. IR: The samples were measured on a Bruker Tensor 27 with an MIR source and DLaTGS as a detector. The instrument was equipped with GladiATR Diamond Crystal Plate from Pike Technologies. Data were collected in the spectral range of 4,000–400 cm^−1^ with a resolution of 4 cm^−1^. Each sample (16 scans) was measured against background (16 scans). The collected data were processed by software Opus (version 6.5), where the atmospheric compensation for water and carbon dioxide, and a baseline correction were made in the interactive mode. EA: Elemental analysis was performed on a Perkin Elmer 2400, series II, CHNS/O analyzer. Solvents were dried by MB-SPS 800, MBraun equipment. [*α*]_D_^20^: Optical rotation was measured on polarimeter Autopol IV by Rudolph Research Analytical (USA). Sodium D doubleline was used, wavelength 589 nm. Measurements were made in chloroform and corrected to 20 °C. UV: Spectra were recorded on spectrometer Specord 210 from Analytic Jena (Germany) in the range 200–700 nm, equipped by software WinAspect. X-ray crystallography: Crystallographic data were collected at 123(2) K on a Nonius KappaCCD diffractometer with graphite monochromated Mo-K_α_ radiation. COLLECT data collection software was utilized and the data were processed with DENZO-SMN [31] and corrected for absorption effects (MULABS [32]). The structures were solved by direct methods (SIR2002 [33] or SHELXS-97 [34]) and refined anisotropically by full matrix least squares on *F* values. Hydrogen atoms were located from the expected geometry and were refined only isotropically. Figures were drawn with Ortep-3 for Windows. A more detailed experimental procedure is described elsewhere [24]. Crystallographic data (excluding structure factors) have been deposited with the Cambridge Crystallographic Data Centre as supplementary publication numbers CCDC 777360-777363, respectively. Copies of the data can be obtained free of charge on application to CCDC, 12 Union Road, Cambridge CB2 1EZ UK. All reagents were of analytical grade and were purchased from regular commercial sources, and were used without any further purification. Probucol (batch no. 087K0759) and all steroid compounds (stigmastanol, cholesterol, β-sitosterol and stigmasterol) were purchased from Sigma-Aldrich, Inc. Silica gel 60 (0.063–0.200 mm) from Merck KgaA was used for column chromatography.

#### 3.1.1. Preparation of Succinobucol [4-{2,6-di-*tert*-butyl-4-[(1-{ [3-*tert*-butyl-4-hydroxy-5-(propan-2-yl)phenyl] sulfanyl}ethyl)sulfanyl]phenoxy}-4-oxobutanoic acid] (Scheme 1)

A nitrogen-purged flask was charged with dry tetrahydrofuran (THF) (30 mL) and potassium *t*-butoxide (0.93 g; 8 mmol) was added into the stirred solvent in three portions. Subsequently, probucol [4,4'-(propane-2,2-diyldisulfanediyl)bis(2,6-di-*tert*-butylphenol)] (**1)** (2.00 g; 3.9 mmol) was added to the cloudy solution and the mixture was stirred for 45 min under a nitrogen atmosphere at ambient temperature, upon which time it turned orange. Succinic acid anhydride was added to the mixture which turned brown, later dark blue [35]. The reaction mixture was stirred overnight, after which it was washed with 2 M NaOH (15 mL), 1 M HCl (20 mL), brine (15 mL), dried over anhydrous Na_2_SO_4_, and the solvent was evaporated under reduced pressure. The crude product was purified by column chromatography in light petroleum ether/diethyl ether with a gradient to diethyl ether, and at the end the silica column was washed with acetone. The obtained fractions were characterized as probucol (0.68 g), succinobucol (**2**) (1.01 g; y = 42%), and the bisester of probucol (0.14 g). Succinobucol was obtained as a white crystalline solid. It was further studied in detail for its polymorphic behavior and the results will be published elsewhere [36]. For atom numbering of succinobucol see Scheme 1. Succinobucol (**2**): *R*_f_ = 0.58 (diethyl ether/light petroleum ether 2:1); mp: 144–145 °C; ^1^H-NMR: *δ* = 1.34 (s, 18H, H-21-23, 25-27), 1.44 (s, 18H, H-29-31,33-35), 1.46 (s, 6H, H-12,13), 2.79 (t, *J* = 6.9 Hz, 2H, 3-H), 2.99 (t, *J* = 6.9 Hz, 2H, H-2), 5.36 (s, 1H, OH-36), 7.45 (s, 2H, H-7,9), 7.63 ppm (s, 2H, H-15,19); ^13^C-NMR: *δ* = 28.4 (C-3), 30.3 (C-29-31,33-35), 30.43 (C-2), 30.59 (C-12,13), 31.45 (C-21-23,25-27), 34.33 (C-28,32), 35.48 (C-20,24), 59.51 (C-11), 122.11 (C-14), 129.62 (C-8), 134.11 (C-15,19), 134.69 (C-7,9), 136.05 (C-16,18), 142.66 (C-6,10), 148.58 (C-5), 155.02 (C-17), 171.87 (C-4), 176.92 (C-1) ppm; ATR IR: *ν*˜ = 3617, 2961, 1752, 1423, 1406, 1366, 1232, 1189, 1155, 1135, 1100, 883 cm^−1^; UV/Vis (acetonitrile): *λ*_max_ (*ε*)=239 (12.12 cm^2^.mg^−1^), 533 nm (2.02 cm^2^·mg^−1^); MS (ESI-TOF) *m*/*z* (%): 279 (44), 379 (40), 640 (100) [*M*+Na]^+^, 656 (48) [*M*+K]^+^; HRMS-FAB *m*/*z* [*M*+Na]^+^ and [*M*+K]^+^ calcd. for C_35_H_52_O_5_S_2_: 639.3154 and 655.2893, found: 639.6405 and 655.6061; Anal. calcd for C_35_H_52_O_5_S_2_: C 68.14, H 8.50, S 10.40, found: C 68.13, H 8.53, S 10.38.

#### 3.1.2. Preparation of Sterol Conjugates of Succinobucol **3–6**

Generally, all conjugates of **2** with stanol/sterols were prepared by Steglich esterification [37]. To a stirred solution of **2** (200 mg; 0.32 mmol) in anhydrous THF (7 mL) was added DMAP (catalytic amount) and a steroid alcohol (plant stanol/sterol or cholesterol in the amount of 0.55 mmol (1.7 equiv. to **2**) or 0.80 mmol (2.5 equiv.); see the specific cases below). DCC (87 mg; 0.42 mmol) was added to the reaction mixture at 0 °C, which was then stirred for 5 min at 0 °C and subsequently overnight at the ambient temperature under nitrogen atmosphere. The precipitated urea was then filtered off and the filtrate was evaporated under reduced pressure. The residue was taken up in diethyl ether and, if necessary, filtered free of any further precipitated urea. The solution was washed twice with 0.5 M HCl and with saturated NaHCO_3_ (aq.), and dried over anhydrous Na_2_SO_4_. The solvent was evaporated under reduced pressure and the final conjugate was isolated from the crude product by column chromatography in diethyl ether/light petroleum ether (40–60 °C) as the mobile phase, at the rate similar to the one used for TLC analyses (see below). For the atom numbering of conjugates **3**–**6** see Scheme 1. 

*Stigmastanol conjugate* **3**: From stigmastanol (0.23 g, 0.55 mmol); the product **3** was obtained as a white crystalline solid (178 mg, y = 55%). Analyses: *R*_f_ = 0.59 (light petroleum ether/diethyl ether 2:1); mp: 141–143 °C; [*α*]_D_^20^=+11.2 (*c*=1.4 μM in CHCl_3_); ^1^H-NMR: *δ* = 0.65 (s, 3H, H-18), 0.82 (s, 3H, H-19), 0.92–0.80 (s, 12H, H-21,26,27,29), 1.34 (s, 18H, H-21-23,25-27), 1.44 (s, 18H, H-29-31,33-35), 1.47 (s, 6H, H-12,13), 2.68 (t, *J* = 7.2 Hz, 2H, H-3), 2.98 (t, *J* = 7.2 Hz, 2H, H-2), 4.73 (m, 1H, H-3’), 5.36 (s, 1H, OH-36), 7.45 (s, 2H, H-7,9), 7.62 ppm (s, 2H, H-15,19); ^13^C-NMR: *δ* = 11.97 (C-19’), 12.06 (C-18’), 12.22 (C-29’), 18.73 (C-21’), 19.03 (C-27’), 19.81 (C-26’), 21.20 (C-11’), 22.62 (C-2’), 23.05 (C-28’), 24.21 (C-15’), 26.07 (C-23’), 27.41 (C-4’), 28.26 (C-16’), 28.59 (C-6’), 29.05 (C-3), 29.14 (C-25), 30.27 (C-29-31,33-35), 30.56 (C-12,13), 30.72 (C-2), 30.72 (C-1’), 31.47 (C-21-23,25-27), 31.98 (C-7’), 33.94 (C-22’), 34.32 (C-28,32), 35.47 (C-20,24), 35.47 (C-8’), 36.16 (C-10’), 36.73 (C-20’), 39.97 (C-12’), 42.58 (C-13’), 44.63 (C-24’), 45.83 (C-5), 54.22 (C-9’), 56.16 (C-17’), 56.41 (C-14’), 59.45 (C-11), 74.38 (C-3’), 122.03 (C-14), 129.43 (C-8), 134.14 (C-15,19), 134.71 (C-7,9), 135.98 (C-16,18), 142.66 (C-6,10), 148.62 (C-5), 155.01 (C-17), 171.47 (C-4), 172.12 (C-1) ppm; ATR IR: *ν* = 3495, 2929, 2855, 2119, 1757, 1717, 1447, 1425, 1362, 1332, 1256, 1174, 1146, 1128, 1100 cm^−1^; UV/Vis (acetonitrile): *λ*_max_ (*ε*) = 202 (11.69 cm^2^·mg^−1^); MS (ESI-TOF) *m*/*z* (%): 431 (100), 445 (48), 667 (8), 1038 (10) [*M*+Na]^+^; HRMS-FAB *m*/*z* [*M*+Na]^+^ calcd for C_64_H_102_O_5_S_2_: 1037.7067, found: 1037.6096; Anal. calcd for C_64_H_102_O_5_S_2_: C 75.69, H 10.12, S 6.31, found: C 75.68, H 10.13, S 6.32. 

*β-Sitosterol conjugate* **4**: From β-sitosterol (0.34 g, 0.80 mmol); the product **4** was obtained as a white crystalline solid (174 mg, y = 57%). Analyses: *R*_f_ = 0.31 (light petroleum ether/diethyl ether 2:1); mp: 165–167 °C; [*α*]_D_^20^ = −17.3 (*c* = 1.6 μM in CHCl_3_); ^1^H-NMR: *δ* = 0.68 (s, 3H, H-18’), 0.88 (d, 3H, *J* = 1.9 Hz, H-26’), 0.87 (d, 3H, *J* = 2.0 Hz, H-27’), 0.92 (d, 3H, *J* = 6.4 Hz, H-21’), 1.02 (s, 3H, H-19’), 1.34 (s, 9H, H-21-23), 1.35 (s, 9H, H-25-27), 1.45 (s, 18H, H-29-31,33-35), 1.47 (s, 6H, H-12,13), 2.33 (d, *J* = 8.0 Hz, 2H, H-4’), 2.70 (t, *J* = 7.2 Hz, 2H, H-3), 3.00 (t, *J* = 7.4 Hz, 2H, H-2), 4.65 (m, 1H, H-3’), 5.36 (s, 1H, OH-36), 5.37 (s, 1H, H-6’), 7.45 (s, 2H, H-7,9), 7.63 ppm (s, 2H, H-15,19); ^13^C-NMR: *δ* = 11.85 (C-18’), 18.72 (C-21’), 19.30 (C-19’), 21.03 (C-11’), 22.54 (C-27’), 22.79 (C-26’), 23.82 (C-23’), 24.27 (C-15’), 27.71 (C-2’), 27.99 (C-25’), 28.21 (C-16’), 29.11 (C-3), 30.29 (C-29-31,33-35), 30.59 (C-12,13), 30.73 (C-2), 31.48 (C-21-23,25-27), 31.87 (C-7’), 31.90 (C-8’), 34.31 (C-28,32), 35.47 (C-20,24), 35.77 (C-4’), 36.19 (C-22’), 36.59 (C-10’), 36.97 (C-20’), 38.06 (C-1’), 39.52 (C-24’), 39.74 (C-12’), 42.32 (C-13’), 50.06 (C-9’), 56.16 (C-17’), 56.70 (C-14’), 59.47 (C-11), 74.55 (C-3’), 122.12 (C-14), 122.69 (C-6’), 129.51 (C-8), 134.10 (C-15,19), 134.65 (C-7,9), 136.02 (C-16,18), 139.58 (C-5’), 142.67 (C-6,10), 148.62 (C-5), 154.99 (C-17), 171.29 (C-4), 172.07 (C-1) ppm; ATR IR: *ν* = 3592, 2950, 2866, 2119, 1756, 1723, 1466, 1425, 1362, 1325, 1254, 1174, 1146, 1129, 1100 cm^−1^; UV/Vis (acetonitrile): *λ*_max_ (*ε*) = 206 (12.96 cm^2^·mg^−1^); MS (ESI-TOF) *m*/*z* (%): 431 (100), 445 (32), 1008 (37) [*M*+Na]^+^; HRMS-FAB *m*/*z* [*M*+Na]^+^ and [*M*+K]^+^ calcd for C_62_H_96_O_5_S_2_: 1008.6597 and 1023.6336, found: 1007.5692 and 1023.5323; Anal. calcd for C_62_H_96_O_5_S_2_: C 75.56, H 9.82, S 6.51, found: C 75.54, H 9.80, S 6.52.

*Stigmasterol conjugate* **5**: From stigmasterol (0.34 g, 0.80 mmol); the product **5** was obtained as a white crystalline solid (161 mg, y = 53%). Analyses: *R*_f_ = 0.65 (light petroleum ether/diethyl ether 6:1); mp: 129–132 °C; [*α*]_D_^20^ = −18.0 (*c* = 1.7 μM in CHCl_3_); ^1^H-NMR: *δ* = 0.70 (s, 3H, H-18’), 0.80 (d, 3H, *J* = 6.5 Hz, H-27’), 0.81 (t, 3H, *J* = 7.3 Hz, H-29’), 0.85 (d, 3H, *J* = 6.5 Hz, H-26’), 1.02 (s, 3H, H-19’), 1.03 (d, 3H, *J* = 6.2 Hz, H-21’), 1.34 (s, 9H, H-21-23), 1.34 (s, 9H, H-25-27), 1.44 (s, 18H, H-29-31,33-35), 1.47 (s, 6H, H-12,13), 2.33 (d, *J* = 7.9 Hz, 2H, H-4’), 2.70 (t, *J* = 7.1 Hz, 2H, H-3), 3.00 (t, *J* = 7.1 Hz, 2H, H-2), 4.65 (m, 1H, H-3’), 5.02 (dd, 1H, *J* = 8.7 Hz, *J* = 6.6 Hz, 23-H), 5.16 (dd, 1H, *J* = 8.8 Hz, *J* = 6.5 Hz, 22-H), 5.36 (s, 1H, OH-36), 5.37 (s, 1H, H-6’), 7.45 (s, 2H, H-7,9), 7.62 ppm (s, 2H, H-15,19); ^13^C-NMR: *δ* = 12.05 (C-18’), 12.22 (C-29’), 18.99 (C-26’), 19.31 (C-19’), 21.03 (C-11’), 21.06 (C-27’), 21.21 (C-21’), 24.36 (C-15’), 25.39 (C-28’), 27.72 (C-2’), 28.88 (C-16’), 29.13 (C-3), 30.30 (C-29-31,33-35), 30.60 (C-12,13), 30.75 (C-2), 31.49 (C-21-23,25-27), 31.88 (C-7’), 31.88 (C-8’), 31.89 (C-25’), 34.32 (C-28,32), 35.48 (C-20,24), 36.62 (C-10’), 36.98 (C-1’), 38.08 (C-4’), 39.65 (C-12’), 40.45 (C-20’), 42.23 (C-13’), 50.09 (C-9’), 51.24 (C-24’), 55.99 (C-17’), 56.81 (C-14’), 59.48 (C-11), 74.56 (C-3’), 122.13 (C-14), 122.70 (C-6’), 129.33 (C-23’), 129.52 (C-8), 134.12 (C-15,19), 134.67 (C-7,9), 136.03 (C-16,18), 138.28 (C-22’), 139.60 (C-5’), 142.69 (C-6,10), 148.64 (C-5), 155.01 (C-17), 171.32 (C-4), 172.08 (C-1) ppm; ATR IR: *ν* = 3627, 2955, 2868, 2119, 1761, 1737, 1420, 1361, 1175, 1149, 1100 cm^−1^; UV/Vis (acetonitrile): *λ*_max_ (*ε*) = 202 (8.44 cm^2^·mg^−1^); MS (ESI-TOF) *m*/*z* (%): 431 (100), 1034 (10) [*M*+Na]^+^; HRMS-FAB *m*/*z* [*M*+Na]^+^ and [*M*+K]^+^ calcd for C_64_H_98_O_5_S_2_: 1033.6753 and 1049.6493, found: 1033.7561 and 1049.7605; Anal. calcd for C_64_H_98_O_5_S_2_: C 75.99, H 9.76, S 6.34, found: C 75.96, H 9.73, S 6.36.

*Cholesterol conjugate* **6**: From cholesterol (0.31 g, 0.80 mmol); the product **6** was obtained as a white crystalline solid (209 mg, y = 71%). Analyses: *R*_f_ = 0.66 (light petroleum ether/diethyl ether 6:1); mp: 166–168 °C; [*α*]_D_^20^ = −12.8 (*c* = 1.5 μM in CHCl_3_); ^1^H-NMR: *δ* = 0.68 (s, 3H, H-18’), 0.94–0.80 (s, 12H, H-21’,26’,27’,29’), 1.02 (s, 3H, H-19’), 1.34 (s, 18H, H-21-23,25-27), 1.44 (s, 18H, H-29-31,33-35), 1.47 (s, 6H, H-12,13), 2.33 (s, 2H, H-4’), 2.70 (t, *J* = 7.0 Hz, 2H, H-3), 3.00 (t, *J* = 7.0 Hz, 2H, H-2), 4.65 (m, 1H, H-3’), 5.36 (s, 1H, H-6’), 5.37 (s, 1H, OH-36), 7.45 (s, 2H, H-7,9), 7.62 ppm (s, 2H, H-15,19); ^13^C-NMR: *δ* = 11.83 (C-18’), 11.96 (C-29’), 18.75 (C-21’), 19.01 (C-27’), 19.29 (C-19’), 19.80 (C-26’), 20.99 (C-11’), 23.04 (C-28’), 24.28 (C-15’), 26.04 (C-23’), 27.67 (C-2’), 28.22 (C-16’), 29.11 (C-3), 29.69 (C-25’), 30.26 (C-29-31,33-35), 30.54 (C-12,13), 30.72 (C-2), 31.45 (C-21-23,25-27), 31.82 (C-7’), 31.89 (C-8’), 33.91 (C-22’), 34.30 (C-28,32), 35.45 (C-20,24), 36.13 (C-4’), 36.56 (C-20’), 36.96 (C-10’), 38.04 (C-1’), 39.69 (C-12’), 42.29 (C-13’), 45.81 (C-24’), 49.99 (C-9’), 56.00 (C-17’), 56.66 (C-14’), 59.43 (C-11), 74.53 (C-3’), 121.99 (C-6’), 122.70 (C-14), 129.40 (C-8), 134.12 (C-15,19), 134.69 (C-7,9), 135.95 (C-16,18), 139.55 (C-5’), 142.64 (C-6,10), 148.60 (C-5), 154.99 (C-17), 171.33 (C-4), 172.11 (C-1) ppm; ATR IR: *ν* = 3500, 2955, 2870, 1757, 1720, 1467, 1326, 1255, 1173, 1146, 1128, 1100 cm^−1^; UV/Vis (acetonitrile): *λ*_max_ (*ε*) = 203 (12.32 cm^2^·mg^−1^); MS (ESI-TOF) *m*/*z* (%): 431 (100), 445 (32), 1036 (20) [*M*+Na]^+^; HRMS-FAB *m*/*z* [*M*+Na]^+^ calcd for C_64_H_100_O_5_S_2_: 1035.6910, found: 1035.7633; Anal. calcd for C_64_H_100_O_5_S_2_: C 75.84, H 9.94, S 6.33, found: C 75.84, H 9.98, S 6.32. All conjugates were recrystallized from an acetonitrile/diethyl ether mixture and their single crystals were studied in detail by X-ray crystallography [24]. 

### 3.2. Toxicity Test

Mouse fibroblasts the Balb/c 3T3 cell line (No. 86110401) were purchased from ECACC (The European Collection of Cell Cultures, Porton Down, UK). Cryo-preserved cells were taken out of a deep-freezing box and left to warm up for 1 min at the ambient temperature. Subsequently, they were transferred to a 25 cm^3^ bottle with 10 mL of cultivation medium (DMEM, penicillin 100 U mL^−1^, L-glutamine 2 mmol L^−1^, streptomycin 100 mg L^−1^, fetal calf serum 5%, new born calf serum 5%). The cells were kept in an incubator saturated by water vapor at 37 °C and under an atmosphere of 5% CO_2_; the medium was changed every 48–72 h. When a mono-layer was reached, the cells were washed with sterilized PBS (5 mL), released by incubation in a 0.25% solution of trypsin with EDTA (0.5 mL; 2–3 min, 37 °C). 5 mL of the cultivation medium was added and centrifuged (10 min, 1,300 rpm, ambient temperature). The pellet was re-suspended in the cultivation medium (20 mL) and the cells were transferred into a 75 cm^3^ cultivating bottle and further cultivated. When the mono-layer was reached, the cells were washed by sterilized PBS (10 mL) and released by incubation in the 0.25% solution of trypsin with EDTA (1 mL; 2–3 min; 37 °C), and were re-suspended in the cultivation medium (10 mL). The suspension was centrifuged (10 min; 1,300 rpm; ambient temperature). The pellet was re-suspended in the cultivation medium (10 mL) and the cells were finally used for experiments. The cells were used in 5 to 10 passages.

Stock solutions were prepared in ethanol to a concentration of 2% (v/v) of ethanol in the medium. The solutions of the tested compounds were stirred in vortex (2 min) and further treated in an ultrasound bath (5 min) to fully dissolve. Control cells were incubated in the medium with a corresponding volume of ethanol only. The solutions of **1**, **2**, and conjugates **3**–**6** were prepared in ethanol in a concentration range of 7.81–2,000 µmol L^−1^.

Cell concentrations were determined by the trypan blue coloring method. The cells were dissolved in the cultivation medium and seeded in a 96-well plate in a concentration of 3.10^4^ cell mL^−1^ in a 0.2 mL well^−1^. After a 24 h incubation of the culture (Jouan incubator - atmosphere 95% air, 5% CO_2_, saturated water vapor, 37 °C), the cultivation medium was exchanged for a medium without serum containing the tested compounds and this was further incubated for 24 h under the same conditions. After this period, the cell damage was observed by detection of mitochondrial dehydrogenase activity (MTT) test [38], whereby a yellow tetrazolium salt is reduced by mitochondrial dehydrogenases of metabolically active cells into purple water insoluble formazan dye, the concentration of which was determined after dissolution into organic solvent by spectrophotometry at 540 nm. MTT is 3-(4,5-dimethylthiazol-2-yl)-2,5-diphenyltetrazolium bromide, a tetrazole.

The IC_50_ values were calculated from the measured values of the absorbance. Tests were carried out in triplicate. The solutions of **1**, **2** and conjugates **3**–**6** were prepared in a concentration range of 0.16–40 µmol L^−1^ of medium. The cells were washed by aseptic PBS after incubation with the tested compounds and, subsequently, 100 µL of fresh medium without serum and 10 µL of MTT solution (5 mg mL^−1^; PBS) were applied. After 3 h of incubation (37 °C; 5% CO_2_), the medium with MTT was evacuated and 200 µL DMSO with 1% ammonia was added into wells and the plate was shaken. The absorbance was measured at 540 nm.

### 3.3. Antioxidant Activity — DPPH Scavenging Assay * [25,39]*

The scavenging activity was evaluated using the DPPH radical, a stable violet radical with a maximum absorbance at 522 nm that can be reduced to a yellow hydrazine derivative. The decrease in the absorbance corresponds to the scavenging activity of the test compound. Stock solutions of the tested compounds (1–1,000 µmol·L^−1^) were prepared in methanol (sonicated 10–60 min, 50 °C) and checked for possible degradation by TLC. Ascorbic acid was used as a positive control of antioxidant capacity. The reaction mixture contained 2.25 mL of a DPPH solution (44.4 mg L^−1^, methanol) and 0.75 mL of the tested compound solution or methanol alone in the control sample. The samples were kept in a closed dark box to protect them from the light and were taken out for the measurements only. The decrease in the absorbance at 522 nm (experimentally established wavelength) was measured with a spectrophotometer until a constant difference between the absorbance of the sample and the control sample (24 hours). The assay was carried out in triplicate.

## 4. Conclusions

Four different succinobucol-steroid conjugates, derived from a plant stanol (stigmastanol), plant sterols (β-sitosterol and stigmasterol) and an animal sterol (cholesterol), were prepared in good yields by Steglich esterification and fully characterized at their molecular and submolecular level. 

The conjugates, together with parental probucol and succinobucol, were tested for their toxicity on mouse fibroblasts Balb/c 3T3 by the MTT test, where the compounds showed low toxicity at their maximal concentrations. Esterification of succinobucol’s free carboxylic group lowers its toxicity, as observed in the comparison with the conjugates **3–6**.

The tested compounds showed similar or even higher antioxidant activity compared to a standard ascorbic acid in the scavenging of the DPPH radical. This was ascribed to the participation of sulfur atoms in the radical scavenging rather than to an activity of phenolic moieties.

The prepared conjugates, as esters which can resist gastric acidic hydrolysis, are expected to be cleaved by pancreatic non-selective lipases into succinobucol and stanol/sterol in the upper part of the small intestine. The released succinobucol may be incorporated into mixed bile acid salt micelles, transported into the enterohepatic circulation, and further distributed within the body incorporated in lipoproteins. The released free plant stanols or sterols then either block the absorption of cholesterol from intestine or reduce its serum level after absorption by influencing its distribution and metabolism. The absorptive pathway of succinobucol may be multiplied in the case of its cholesterol conjugate.

The development of succinobucol-steroid conjugates, which, on one side, target anti-inflammatory and antioxidant processes, and on the other side, decrease the LDL-cholesterol level, increase the absorption and targeting of the drug into the enterohepatic circulation. This may add to the armamentarium of agents used in the treatment of atherosclerotic diseases and type 2 diabetes. Nevertheless, further biological studies are needed to discover their full medicinal potential.
